# Analysis of the characteristics, efficiency, and influencing factors of third-party mediation mechanisms for resolving medical disputes in public hospitals in China

**DOI:** 10.1186/s12889-024-19366-0

**Published:** 2024-07-09

**Authors:** Yanfei Shen, Gaiyun Li, Zhiguo Tang, Qi Wang, Zurong Zhang, Xiangyong Hao, Xuemei Han

**Affiliations:** 1 Department of Medical Management, Gansu Provincial Hospital, Lanzhou, China; 2 School of Public Health, Lanzhou University, Lanzhou, China; 3 Law School of Lanzhou University, Lanzhou, China; 4 Department of General Surgery, Gansu Provincial Hospital, Lanzhou, China

**Keywords:** Medical disputes, Third-party mediation, Medical liability insurance, Medical mediation, Compensation

## Abstract

**Background:**

Medical disputes, which are prevalent in China, are a growing global public health problem. The Chinese government has proposed third-party mediation (TPM) to resolve this issue. However, the characteristics, efficiency, and influencing factors of TPM in resolving medical disputes in public hospitals in China have yet to be determined.

**Methods:**

We conducted a systematic study using TPM records from medical disputes in Gansu Province in China from 2014 to 2019. A χ2 test was used to compare differences between groups, and binary logistic analysis was performed to determine the factors influencing the choice of TPM for resolving medical disputes.

**Results:**

We analyzed 5,948 TPM records of medical disputes in Gansu Province in China. The number of medical disputes and the amount of compensation awarded in public hospitals in the Gansu Province increased annually from 2014 to 2019, with most of the disputes occurring in secondary and tertiary hospitals. Approximately 89.01% of the medical disputes were handled by TPM; the average compensation amount with TPM was Chinese Yuan (CNY) 48,688.73, significantly less than that awarded via court judgment and judicial mediation. TPM was more likely to succeed in settling medical disputes in the < CNY10,000 compensation group than in the no-compensation group (odds ratio [OR] = 3.14, 95% confidence interval [CI] 1.53–6.45). However, as the compensation amount increased, the likelihood of choosing TPM decreased significantly. Moreover, TPM was less likely to be chosen when medical disputes did not involve death (OR = 0.49, 95% CI 0.36–0.45) or when no-fault liability was determined (vs. medical accidents; OR = 0.37, 95% CI 0.20–0.67).

**Conclusion:**

Our findings demonstrate that TPM mechanisms play a positive role in efficiently reducing compensation amounts and increasing medical dispute resolution rates which was the main settlement method in resolving medical disputes in public hospitals of Gansu Province in China. TPM could help greatly reduce conflicts between doctors and patients, avoid litigation, and save time and costs for both parties. Moreover, compensation amounts, non-fatal outcomes, and no-fault liability determinations influence the choice of TPM for settling medical disputes.

## Background

Medical disputes, very common in China, are a growing global public health problem [1–3]. It has been reported that 42.2%–83.3% of medical staff in China have experienced workplace violence, indicating that medical disputes and tense doctor–patient relationships are frequent [4, 5]. Medical disputes in China most commonly occur in secondary and tertiary hospitals, and the risk for disputes exists in almost all departments [6].

There are many causes of medical disputes. At the hospital or medical worker level, disputes arise from issues such as poor-quality medical services [7], misdiagnosis [8], a lack of doctor–patient communication [9], informed consent infringement [10], overloaded workloads [11], poor hospital management and inappropriate internal incentives [12]. The main aspects contributing to disputes at the patient and family level include increased awareness of rights [7], high expectations for treatment outcomes, high medical costs [13], and a lack of trust in physicians and hospitals [9, 14]. At the government level, disputes arise from a lack of supervision, the financing mechanisms for public hospitals, and the insufficient investment of medical funds [15]. In addition, the media mainly covers inaccurate or negative reports about hospitals and doctors [16]. One particular type of hospital violence, commonly referred to as "*Yinao*" in Chinese, involves organized unemployed gangs paid by patients' families to create medical disputes in the hope of obtaining better compensation from hospitals than that obtained through formal methods [17].

Consequently, the frequent occurrence of medical disputes has weakened doctor–patient trust and led to further deterioration of the medical environment [18–21]. Increased friction between physicians and patients makes physicians more defensive, impacting medical care quality and contributing to rising healthcare expenses [22–24]. Some medical disputes are resolved by medical malpractice lawsuits, causing substantial economic and psychological burdens on medical practitioners [25]. The essence of the doctor–patient relationship is a community of health interests, and a harmonious doctor–patient relationship is key to building a favorable social environment. Thus, it is critical to reasonably settle medical disputes to defend the legitimate interests of both physicians and patients, and foster a positive doctor–patient relationship.

The Chinese government has proposed multiple medical dispute resolution approaches, including negotiation, people’s mediation, administrative mediation, and litigation [26]. Regarding people's mediation for medical disputes, the doctors and patients jointly apply to a third-party mediation (TPM) institution, the People’s Mediation Committee for Medical Disputes [26]. The TPM mechanism is flexible, allowing active coordination between the hospital and the patient and ultimately helping both parties reach an agreement; this approach has been adopted in many provinces in China [27–29].

However, research on medical dispute resolution methods has focused more on litigation [29–35]; some studies have emphasized the role of mediation, but research into the characteristics, efficiency, and influencing factors of TPM in resolving medical disputes using a large number of detailed cases is lacking [19, 27, 29, 36]. Therefore, we analyzed TPM practices using data from 5,948 cases in Chinese public hospitals from 2014 to 2019 in Gansu, a western province in China. First, this study provided a comprehensive understanding of the detailed characteristics and processes of TPM in China. Second, the efficiency of TPM and the factors influencing the choice of TPM were clarified. Thus, our findings could act as a reference and the basis for policy recommendations for public hospitals, establishing a sound medical dispute resolution mechanism and a reasonable medical dispute risk management strategy.

## Methods

### Hospitals included in this study

As an important hub of the Belt and Road Economic Belt in western China, Gansu is one of the pilot areas for medical reform in the country. According to the 2020 Statistical Bulletin of Health Development, there are 26,250 medical and health institutions in Gansu Province, including 288 public medical institutions, and the total number of visits to medical and health institutions was ~ 127.326 million, with an absolute value of CNY3531.72 for per capita health costs [37]. The total health expenditure of Gansu Province was CNY93.501 billion, accounting for 10.72% of the Gross Domestic Product (GDP) [37]. Therefore, our research was conducted on public hospitals in the Gansu Province that participated in TPM for medical disputes.

### Mediation of medical disputes in Gansu Province

**Fig. 1:**
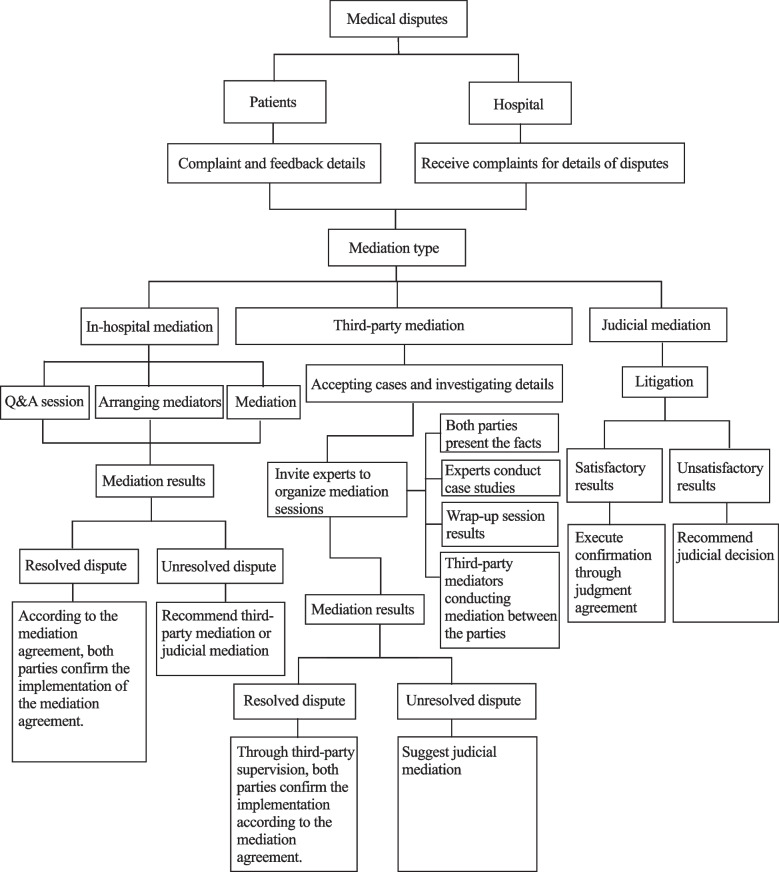
Mediation process for medical disputes in Gansu Province

Medical disputes can be resolved from either the medical side or the patient side through voluntary negotiation, TPM, administrative mediation, lawsuits in the people's court, and other channels dictated by laws and regulations [26]. This study focused on the three main ways of resolving medical disputes in Gansu Province: voluntary negotiation between the two parties (in-hospital mediation), TPM, and judicial mediation. The detailed mediation process for medical disputes in Gansu Province is shown in Fig. 1.

### Data sources

We used data from the Gansu Provincial TPM committee from January 1, 2014, to December 31, 2019, which contains detailed information on case-level medical disputes. The inclusion criteria were as follows: (1) medical disputes occurring in a public hospital in Gansu Province in China that (2) were resolved by TPM. No restrictions were placed on the case origin or the age, sex, and ethnicity of the patients. Cases with important information missing, such as hospital category, specialty, survival outcome, responsibility determination, resolution, etc., were excluded.

### Data extraction

Two researchers (Y-F S and G-Y L) independently collected and cross-checked the extracted data from the cases that met the inclusion and exclusion criteria according to the self-designed data extraction form. The contents of the extracted data are listed in the following table. We contacted the corresponding hospitals to obtain additional information for incomplete case reports.

### Statistical analysis

Microsoft Excel worksheet was used to collate data, and Stata 15.0 was used for statistical analyses. Percentages were used to indicate the proportion of events, a χ2 test was used to compare differences between groups, and binary logistic analysis was performed to determine the factors influencing the choice of TPM for resolving medical disputes. All analyses were performed with a two-sided test, and *P* < 0.05 indicated significance.

## Results

### Basic characteristics of the included medical dispute cases

In all, 5,948 cases from January 1, 2014, to December 31, 2019, from the Gansu Provincial TPM committee, were included. As shown in Table 1, the number of medical disputes in public hospitals increased yearly from 2014 to 2019, and disputes were most frequent in secondary and tertiary hospitals. Moreover, surgery had the highest number of occurrences (42.50%), followed by obstetrics and gynecology (22.75%) and internal medicine (17.99%). In addition, 2,394 (40.25%) of the medical disputes involved deaths. The main liability determination was fault liability (35.05%), followed by technical fault (22.98%) and no fault (14.07%). The main settlement method was TPM (81.81%), followed by withdrawal (7.20%) and judicial decision (6.66%), and the success rate of TPM in public hospitals of Gansu Province was 89.01% (TPM and withdrawal cases are considered successful TPM cases).

**Table 1:** Basic characteristics of the included medical dispute cases in public hospitals of Gansu Province

Variable	All (*n* = 5948)	Variable	All (*n* = 5948)
**Years**		Otolaryngology	66 (1.11%)
2014	706 (11.87%)	Other	62 (1.04%)
2015	863 (14.51%)	**Death**	
2016	988 (16.61%)	Yes	2394 (40.25%)
2017	1029 (17.30%)	No	3554 (59.75%)
2018	1185 (19.92%)	**Liability determination**	
2019	1177 (19.79%)	Medical accidents	547 (9.20%)
**Hospital category**		Informed consent	331 (5.56%)
Primary	370 (6.22%)	Technical fault	1367 (22.98%)
Secondary	3118 (52.42%)	No fault	837 (14.07%)
Tertiary	2460 (41.36%)	Poorly defined	583 (9.80%)
**Sex**		Mismanagement	198 (3.33%)
Male	2796 (47.01%)	Fault of responsibility	2085 (35.05%)
Female	3152 (52.99%)	**Resolution methods**	
**Specialty**		Judicial decision	396 (6.66%)
Internal medicine	1070 (17.99%)	Judicial mediation	87 (1.46%)
Surgery	2528 (42.50%)	Withdrawal of cases	428 (7.20%)
Obstetrics and gynecology	1353 (22.75%)	Third-party mediation	4866 (81.81%)
Pediatrics	393 (6.61%)	Open cases	171 (2.87%)
Traditional Chinese medicine	98 (1.65%)	**Age (years)**	
Dentistry	57 (0.96%)	< 5	872 (14.66%)
Emergency medicine	51 (0.86%)	5 ~ 24	544 (9.15%)
Medical imaging	105 (1.77%)	25 ~ 44	1523 (25.61%)
Ophthalmology	118 (1.98%)	45 ~ 64	2034 (34.20%)
Psychiatry	47 (0.79%)	≥ 65	975 (16.39%)

### Characteristics of compensation of the included medical dispute cases

**Fig. 2:**
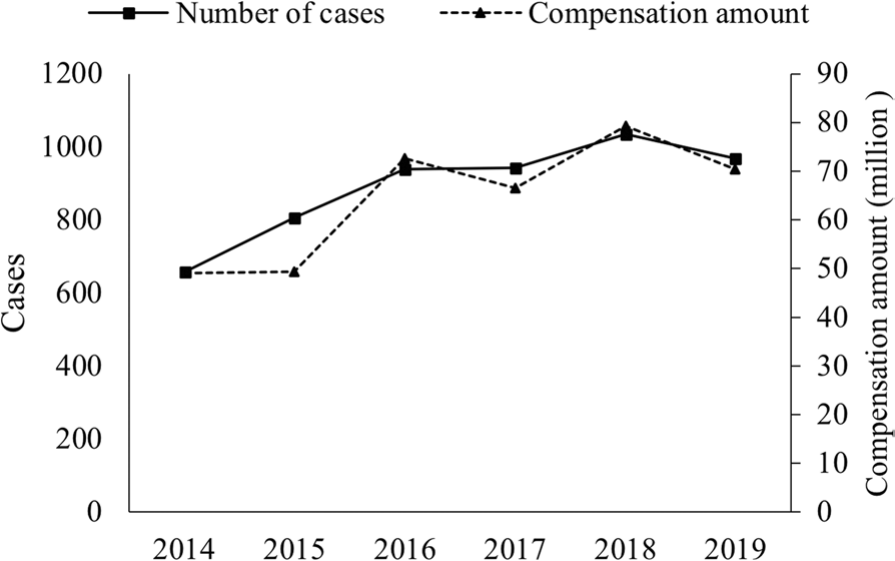
Number of medical disputes and amount of compensation from 2014 to 2019

**Fig. 3:**
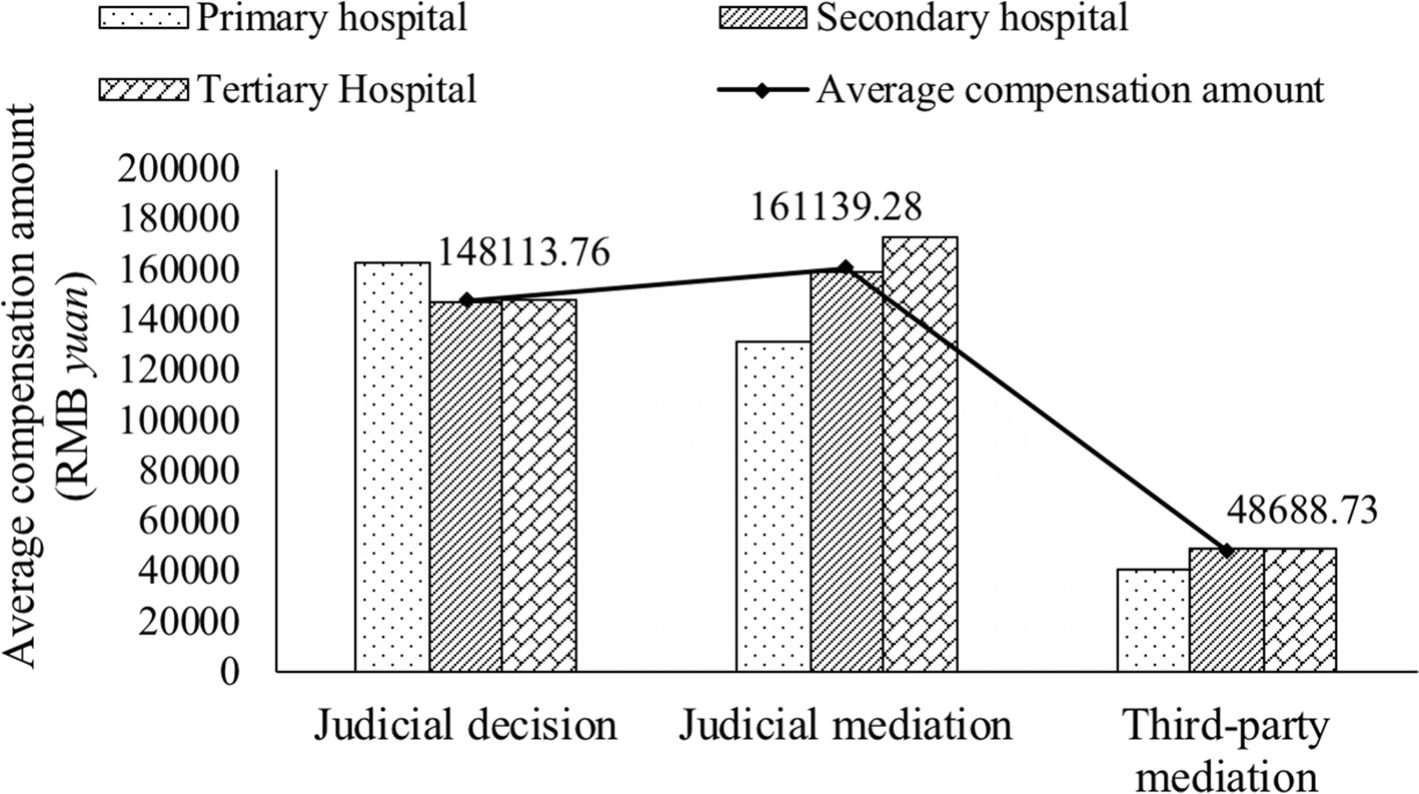
Compensation among medical institutions and settlement methods

Because the compensation was not recorded for open and withdrawn cases (599 cases), 5,349 cases were included in the analysis of the characteristics of compensation of the included medical dispute cases. As shown in Fig. 2, the overall number of medical dispute cases and the amount of compensation showed an increasing trend from 2014 to 2019, with the number of medical dispute cases increasing from 656 in 2014 to 969 in 2019 and the amount of compensation increasing from CNY 49.15 million in 2014 to CNY 70.36 million in 2019. As shown in Fig. 3, TPM's average compensation sum of CNY 48,688.73 is much less than the CNY 148,113.76 and CNY 161,139.28 awarded via judicial decision and judicial mediation, respectively. The average compensation amount regarding primary hospitals in judicial judgment reached CNY 163,446.55, while that regarding tertiary hospitals in judicial mediation was CNY 172,801.36. Moreover, by TPM, the average compensation amount was lowest for primary hospitals (CNY 40,731.85), and little difference was observed between secondary (CNY 49,184.63) and tertiary (CNY 49,322.73) hospitals.

### Possible influencing factors regarding the choice of TPM for settling medical disputes

As shown in Table 2, the year, patient sex, department, liability assessment opinion, and compensation amount were factors possibly influencing whether to choose TPM for settling medical disputes (*P* < 0.05), while the hospital category, whether death occurred, and age were not statistically significant between the two groups (*P* > 0.05).

**Table 2:** Possible influencing factors on whether to choose TPM in the settlement of medical disputes

Variable	Whether to choose TPM		*χ*^*2*^	*P*
	**No**	**Yes**		
**Years**			28.62	0.000
2014	33 (6.8%)	623 (12.8%)		
2015	63 (13.0%)	742 (15.2%)		
2016	85 (17.6%)	855 (17.6%)		
2017	82 (17.0%)	862 (17.7%)		
2018	128 (26.5%)	907 (18.6%)		
2019	92 (19.0%)	877 (18.0%)		
**Hospital category**			4.86	0.088
Primary	21 (4.3%)	318 (6.5%)		
Secondary	247 (51.1%)	2557 (52.3%)		
Tertiary	215 (44.5%)	1991 (40.9%)		
**Sex**				
Male	254 (52.6%)	2253 (46.3%)	6.97	0.008
Female	229 (47.4%)	2613 (53.7%)		
**Specialty**			20.80	0.035
Internal medicine	80 (16.6%)	880 (18.1%)		
Surgery	240 (49.7%)	2057 (42.3%)		
Obstetrics and gynecology	104 (21.5%)	1122 (23.1%)		
Pediatrics	18 (3.7%)	324 (6.7%)		
Traditional Chinese medicine	4 (0.8%)	88 (1.8%)		
Dentistry	3 (0.6%)	48 (1.0%)		
Emergency medicine	4 (0.8%)	43 (0.9%)		
Medical imaging	8 (1.7%)	78 (1.6%)		
Ophthalmology	12 (3.7%)	92 (1.9%)		
Psychiatry	2 (0.4%)	34 (0.7%)		
Otolaryngology	7 (1.4%)	50 (1.0%)		
Other	1 (0.2%)	50 (1.0%)		
**Death**				
Yes	188 (38.9%)	2063 (42.4%)	2.17	0.140
No	295 (61.1%)	2803 (57.6%)		
**Liability determination**			2200.00	0.000
Medical accidents	24 (5.0%)	495 (10.2%)		
Informed consent	14 (2..9%)	310 (6.4%)		
Technical fault	70 (14.5%)	1253 (25.8%)		
No fault	42 (8.7%)	692 (14.2%)		
Poorly defined	223 (46.2%)	10 (0.2%)		
Mismanagement	9 (1.9%)	184 (3.8%)		
Fault of responsibility	101 (20.9%)	1922 (39.5%)		
**Compensation amount (CNY)**			508.65	0.000
0	53 (11.0%)	680 (14.0%)		
> 0, ≤ 10,000	19 (3.9%)	1266 (26.0%)		
> 10,000, ≤ 50,000	69 (14.3%)	1570 (32.3%)		
> 50,000, ≤ 100,000	91 (18.8%)	667 (13.7%)		
> 100,000	251 (52.0%)	683 (14.0%)		
**Age (years)**			5.12	0.275
< 5	65 (13.5%)	701 (14.4%)		
5 ~ 24	51 (10.6%)	434 (8.9%)		
25 ~ 44	120 (24.8%)	1248 (25.6%)		
45 ~ 64	180 (37.3%)	1666 (34.2%)		
≥ 65	67 (13.9%)	817 (16.8%)		

### Binary logistic analysis of the factors influencing the choice of TPM for settling medical disputes

As shown in Table 3, the binary logistic regression analysis showed no significant difference in hospital category, patient sex, hospital department, or patient age regarding the choice of TPM for settling medical disputes (*P* > 0.05). TPM was more likely to succeed in settling medical disputes in the < CNY10,000 compensation group than in the no-compensation group (odds ratio [OR] = 3.14, 95% confidence interval [CI] 1.53–6.45). However, as the compensation amount increased, the likelihood of choosing TPM decreased significantly; for example, between the CNY50,000 compensation group and the CNY100,000 (OR = 0.24, 95% CI 0.14–0.42) and > CNY100,000 (OR = 0.08, 95% CI 0.05–0.14) compensation groups. Moreover, TPM was less likely to be chosen when medical disputes did not involve death (OR = 0.49, 95% CI 0.36–0.45) or when no-fault liability was determined (vs. medical accidents; OR = 0.37, 95% CI 0.20–0.67).

**Table 3:** Binary logistic analysis of factors influencing the choice of TPM for settling medical disputes

Influencing Factors	Category	Std. Dev	*P*	OR (95% CI)
**Compensation amount (CNY)**	0			*Ref* (1.00)
	> 0, ≤ 10,000	1.15	0.002	3.14 (1.53, 6.45)
	> 10,000, ≤ 50,000	0.26	0.684	0.89 (0.50, 1.57)
	> 50,000, ≤ 100,000	0.07	0.000	0.24 (0.14, 0.42)
	> 100,000	0.02	0.000	0.08 (0.05, 0.14)
**Hospital category**	Primary			*Ref* (1.00)
	Secondary	0.28	0.630	0.86 (0.45, 1.61)
	Tertiary	0.09	0.708	0.88 (0.46, 1.68)
**Sex**	Male			*Ref* (1.00)
	Female	0.16	0.390	1.13 (0.86, 1.49)
**Specialty**	Internal medicine			*Ref* (1.00)
	Surgery	0.16	0.240	0.79 (0.53, 1.17)
	Obstetrics and gynecology	0.22	0.585	0.87 (0.53, 1.44)
	Pediatrics	0.68	0.200	1.68 (0.76, 3.71)
	Traditional Chinese medicine	2.27	0.262	2.63 (0.49, 14.24)
	Dentistry	0.35	0.315	0.47 (0.11, 2.05)
	Emergency medicine	0.77	0.851	0.84 (0.14, 5.11)
	Medical imaging	1.00	0.621	1.42 (0.36, 5.66)
	Ophthalmology	0.45	0.798	0.88 (0.32, 2.38)
	Psychiatry	0.73	0.925	0.93 (0.20, 4.31)
	Otolaryngology	1.24	0.665	1.45 (0.27, 7.77)
	Other	49.83	0.244	19.55 (0.13, 2892.87)
**Death**	Yes			*Ref* (1.00)
	No	0.07	0.000	0.49 (0.36, 0.65)
**Liability determination**	Medical accidents			*Ref* (1.00)
	Informed consent	0.21	0.129	0.57 (0.28, 1.18)
	Technical fault	0.22	0.589	0.87 (0.53, 1.44)
	No fault	0.11	0.001	0.37 (0.20, 0.67)
	Poorly defined	0.00	0.000	0.00 (0.00, 0.00)
	Mismanagement	0.24	0.168	0.55 (0.24, 1.28)
	Fault of responsibility	0.20	0.398	0.81 (0.50, 1.32)
**Age**		0.38	0.705	1.00 (0.99, 1.01)
**Years**	2014			*Ref* (1.00)
	2015	0.28	0.11	0.64 (0.37, 1.11)
	2016	0.28	0.28	0.74 (0.43, 1.27)
	2017	0.26	0.003	0.46 (0.27, 0.76)
	2018	0.26	0.002	0.45 (0.27, 0.74)
	2019	0.27	0.86	0.62 (0.36, 1.07)

## Discussion

This study focused on the characteristics of medical disputes in public hospitals in China, clarified the efficiency of TPM, and assessed the factors influencing the choice of TPM for settling medical disputes. The results showed an annual increase in medical disputes in public hospitals in Gansu Province from 2014 to 2019, consistent with the general sharp increase in medical disputes in China over the last decade [38] and highlighting the strained doctor–patient relationship and the need to improve the medical environment in China. However, TPM represented a sound medical dispute resolution mechanism, with a success rate of 89.01% in public hospitals in Gansu Province; additionally, the average compensation amount awarded under the TPM mechanism was significantly less than that awarded through court judgment and judicial mediation. Moreover, the choice of TPM for settling medical disputes was influenced by the compensation amount, whether the medical disputes involved death, and whether no-fault liability was determined.

Therefore, TPM plays a positive role in efficiently reducing compensation amounts and increasing medical dispute resolution rates. TPM can provide an opportunity for doctors and patients to talk, negotiate, and apologize, moving medical disputes outside the hospital for resolution, protecting the operational order of the hospital and improving the doctor–patient relationship. Compared with judicial mediation and judicial decision, TPM can reduce compensation, litigation costs, and attorney fees; additionally, TPM can reduce the incidence of *Yinao* in exchange for higher compensation. Wang et al. demonstrated that mediation might significantly minimize doctor–patient conflict and avert litigation, saving time and money [28]. TPM committees for medical disputes serve the interests of physicians and patients and assist the government in resolving social issues, preventing hospital–patient confrontations, and preventing disputes from escalating [27]. With advances in medical technology, some patients have unrealistic expectations for treatment outcomes, and physicians are often asked to respond to any outcome that falls short of the patient's expectations [39]. This phenomenon can be addressed by communicating with patients and their families through TPM to increase their understanding of the limitations and unknowns in medicine, reduce medical disputes, and disclose to patients promptly that medical errors and complications are associated with lower litigation rates [40], thus reducing litigation rates, easing the pressure on doctors, and reducing defensive medicine.

This study showed that the high-risk departments for medical disputes in public hospitals in Gansu Province were surgery, obstetrics and gynecology, and internal medicine, with the highest number of disputes associated with surgeries (42.50%). Previous studies have also shown that the high-risk departments for medical disputes are mainly obstetrics and gynecology [41], surgery [42], internal medicine, and emergency medicine [43]. It has been reported that the amount of compensation awarded in medical disputes depends on the specialty department, with surgery facing the highest risk, followed by obstetrics and gynecology [44]. Therefore, the handling of medical disputes in surgical departments requires extra attention, and as surgical medical staff, we must continue to pay high attention to the perioperative period and improve the corresponding medical management system. Likewise, in an era of increasing medical malpractice litigation, medical personnel must be aware of the basic legal concepts of medical malpractice to avoid unnecessary medical disputes [45].

A medical risk-sharing mechanism could be introduced for departments with high medical risk, such as surgery or obstetrics and gynecology. Medical risk-sharing mechanisms comprise a combination of medical liability insurance, medical risk funds, physician liability insurance, and surgical accident insurance. Medical liability insurance is generally purchased by the hospital. Medical risk funds are money set aside from medical expenses to pay the expenses incurred by the hospital in the event of a medical accident after clarifying their responsibilities through a third-party appraisal agency or mediation agency.

Among the medical disputes in public hospitals in Gansu Province, 35.05% were due to the fault of medical personnel, and 22.98% were due to technical failures. Most medical disputes in Gansu Province public hospitals can be avoided actively. This suggests that hospitals need to further improve employees' professional skills. In addition, the number of clinical visits is a key determinant of physician malpractice risk; the higher the number of visits is, the higher the annual risk for physicians will be [46]. Therefore, the hospital should be equipped with sufficient medical staff to avoid medical errors due to doctors’ high workload.

Another major finding of this study is that the average compensation amount awarded via TPM is much smaller than that awarded via judicial judgment and judicial mediation, indicating that TPM not only has a positive effect on easing the relationship between doctors and patients and safeguarding the legitimate rights and interests of both doctors and patients but also helps build a low-cost medical and health service system and a low-cost and high-efficiency medical dispute resolution mechanism.

Moreover, whether TPM is chosen for the settlement of medical disputes is influenced by the compensation amount, whether death occurred, and whether no-fault liability was determined. The greater the expected compensation amount is, the less likely it is that TPM will be chosen; i.e., the higher the compensation amount claimed, the less likely the dispute is to be resolved through mediation. Less serious cases are more likely than fatal cases to be resolved through mediation and yield a lower compensation amount at settlement. This finding indicates that patients correctly understand the seriousness of the consequences of medical care [28].

In addition, whichever path of medical dispute resolution is chosen, the patient's experience and feelings must be fully considered, including any negative consequences that may arise. The participant was exposed to the secondary psychological distress of the medical profession, the lawyer and the legal profession in the specific situation of the medical dispute, and the third psychological distress of living as a disabled person [47]. And these are based on the patient's own feelings or experiences, which is more conducive to establishing the direction of psychological and social intervention for the medical accident based on the understanding of the substantial experiences of the victims of the medical accident. Patient safety incidents are also considered to be important factors influencing the choice of medical dispute mediation, and the positive influence of patients, family members or medical personnel on patient safety incidents will significantly reduce or avoid the occurrence of patient medical disputes [48]. Therefore, healthcare institutions should actively pay attention to and promote the frequency and content of patient safety incident reporting, and avoid the occurrence of medical disputes as soon as possible.

This study has some limitations. First, due to the availability of data, our analysis mainly focused on cases in Gansu Province, and further analyses could include data from other provinces to confirm whether our findings are applicable at the national level. Second, there is a potential bias in the data sources because some medical disputes in public hospitals are resolved privately by hospitals or doctors; thus, these cases are not recorded by TPM committees and may have been missed. Third, we did not collect the data on medical disputes arising from different types of surgeries, side effects, complications, sequelae, the degree of harm caused by the patient safety incident, hospitalization days, quality of post-care nursing staff, which limited further investigation of the relationships of TPM mechanisms with compensation amounts and medical dispute resolution rates and our subsequent studies will focus on these issues.

## Conclusions

TPM plays a positive role in efficiently reducing compensation amounts and increasing medical dispute resolution rates which was the main settlement method in resolving medical disputes in public hospitals in Gansu Province in China. TPM could help greatly reduce conflicts between doctors and patients, avoid litigation, and save time and costs for both parties. Moreover, compensation amounts, non-fatal outcomes, and no-fault liability determinations influence the choice of TPM for settling medical disputes.

## Data Availability

The data that support the findings of this study are available from Gansu provincial TPM committee but restrictions apply to the availability of these data, which were used under license for the current study, and so are not publicly available. Data are however can be obtained from Lutang Li, director of the Gansu TPM Committee, Tel.: + 86–9,314,539,801.

## Abbreviations

TPM: Third-party mediation
CNY: Chinese Yuan
GDP: Gross domestic product
OR: Odds ratio
CI: Confidence interval
